# Advancements in the Protocol for Rate of Force Development/Relaxation Scaling Factor Evaluation

**DOI:** 10.3389/fnhum.2021.654443

**Published:** 2021-03-29

**Authors:** Darjan Smajla, Jure Žitnik, Nejc Šarabon

**Affiliations:** ^1^Faculty of Health Sciences, University of Primorska, Izola, Slovenia; ^2^Human Health Department, InnoRenew CoE, Izola, Slovenia; ^3^Andrej Marušič Institute, University of Primorska, Koper, Slovenia; ^4^S2P, Science to Practice, Ltd., Laboratory for Motor Control and Motor Behavior, Ljubljana, Slovenia

**Keywords:** muscle, explosive strength, regulation, testing, assessment, rate of torque development

## Abstract

Brief submaximal actions are important for wide range of functional movements. Until now, rate of force development and relaxation scaling factor (RFD-SF and RFR-SF) have been used for neuromuscular assessment using 100–120 isometric pulses which requires a high level of attention from the participant and may be influenced by physiological and/or psychological fatigue. All previous studies have been conducted on a smaller number of participants which calls into question the eligibility of some of the outcome measures reported to date. Our aims were: (1) to find the smallest number of rapid isometric force pulses at different force amplitudes is still valid and reliable for RFD-SF slope (k_*R*__*F*__*D*__–SF_) and RFR-SF slope (k_*RFR–SF*_) calculation, (2) to introduce a new outcome measure – theoretical peak of rate of force development/relaxation (TP_*RFD*_ and TP_*RFR*_) and (3) to investigate differences and associations between k_*RFD–SF*_ and k_*RFR–SF*_. A cross-sectional study was conducted on a group of young healthy participants; 40 in the reliability study and 336 in the comparison/association study. We investigated the smallest number of rapid isometric pulses for knee extensors that still provides excellent reliability of the calculated k_*RFD–SF*_ and k_*RFR–SF*_ (ICC_2_,_1_ ≥ 0.95, CV < 5%). Our results showed excellent reliability of the reduced protocol when 36 pulses (nine for each of the four intensity ranges) were used for the calculations of k_*RFD–SF*_ and k_*RFR–SF*_. We confirmed the negligibility of the y-intercepts and confirmed the reliability of the newly introduced TP_*RFD*_ and TP_*RFR*_. Large negative associations were found between k_*RFD–SF*_ and k_*RFR–SF*_ (*r* = 0.502, *p* < 0.001), while comparison of the absolute values showed a significantly higher k_*RFD–SF*_ (8.86 ± 1.0/s) compared to k_*RFR–SF*_ (8.03 ± 1.3/s) (*p* < 0.001). The advantage of the reduced protocol (4 intensities × 9 pulses = 36 pulses) is the shorter assessment time and the reduction of possible influence of fatigue. In addition, the introduction of TP_*RFD*_ and TP_*RFR*_ as an outcome measure provides valuable information about the participant’s maximal theoretical RFD/RFR capacity. This can be useful for the assessment of maximal capacity in people with various impairments or pain problems.

## Introduction

The use of the rate force development scaling factor (RFD-SF) to assess rapid force generation has been more frequently performed and reported after the protocol verification by Bellumori et al. (2011). Since then RFD-SF protocol has been used for isometric neuromuscular assessment of different muscle groups (Casartelli et al., 2014; Djordjevic and Uygur, 2017), to explore the effects of aging (Bellumori et al., 2013) and different diseases such as osteoarthritis (Šarabon et al., 2020a), and in studies exploring lateral asymmetries in different sports (Boccia et al., 2018; Smajla et al., 2020). Moreover, some studies have shown that rate of force relaxation scaling factor (RFR-SF) can be assessed using the RFD-SF protocol with the aim to evaluate the ability of quick relaxation of submaximal muscle forces (Mathern et al., 2019). This ability was shown to be impaired in people with knee osteoarthritis (Šarabon et al., 2020a) and multiple sclerosis (Uygur et al., 2020).

In the verified methodological protocols, maximal voluntary contraction (MVC) is performed prior to RFD-SF/RFR-SF testing to determine the greatest value of force during the performed task (Bellumori et al., 2011; Mathern et al., 2019). Force values corresponding to different submaximal percentages of the maximal force (%MVC) are then calculated and displayed as visual references when the subject performs the task repetitions (RFD-SF/RFR-SF protocol) (Bellumori et al., 2011; Djordjevic and Uygur, 2017; Mathern et al., 2019). Previous methodological studies suggest at least 120 brief isometric force pulses at different force amplitudes (%MVC) to be performed for a valid calculation of RFD-SF related measures (Bellumori et al., 2011; Djordjevic and Uygur, 2017). A similar protocol has been suggested for RFR-SF related measures (Mathern et al., 2019). The regression parameters are then calculated from the relationship between peak force and corresponding peak RFD and peak RFR. The slopes of these relationships (k_*RFD–SF*_ and k_*RFR–SF*_) quantify the magnitude of the linear relationship between peak force and respective RFD/RFR. From the same regression, r^2^ is obtained, revealing the consistency of the RFD/RFR scaling with the peak force of the submaximal force pulse (Bellumori et al., 2011; Mathern et al., 2019). Moreover, it is possible to calculate y-intercept of the regression lines which was shown to be unreliable in the previous literature (Casartelli et al., 2014; Bellumori et al., 2017; Djordjevic and Uygur, 2017). Additional outcome measure can be calculated from the regression line equation (*y* = *k* × *x* + *n*). Based on each individual regression line, we can calculate theoretical (when *x* = 100) peak RFD or RFR (TP_*RFD*_, TP_*RFR*_) for each participant which can represent more valuable information about someone’s neuromuscular ability. This outcome measure has not been previously evaluated and is introduced for the first time in this study along with the reliability evaluation of this outcome measure.

Previously described RFD-SF/RFR-SF protocol also requires participants to be attentive for extended periods (4–5 subsets with 25 isometric pulses every 3–4 s and 60 s between each subset), which is likely to have an influence on outcome results. This raises the question whether a shorter protocol for this type of neuromuscular assessment would yield similar results. In the RFD-SF assessment of a dynamic task (drop jumps) authors have shown that a reliable and linear k_*RFD–SF*_ calculation can be obtained by performing fewer number of repetitions. Specifically, it was shown that k_*RFD–SF*_ of a dynamic multi-joint task assessed with 60 drop jumps provides acceptable reliability and linear relationship (Šarabon et al., 2020b). Moreover, the adapted protocol for RFD-SF and RFR-SF measurement using lower amplitude of submaximal forces (20–60 %MVC) with the aim to avoid pain in knee osteoarthritis patients has been shown to have acceptable reliability despite the omission of the highest amplitude submaximal force when calculating k_*RFD–SF*_ and k_*RFR–SF*_ (Šarabon et al., 2020a). These findings raised questions regarding previously proposed RFD-SF and RFR-SF protocols under isometric conditions (Bellumori et al., 2011; Djordjevic and Uygur, 2017; Mathern et al., 2019), specifically, the number of force pulses required to still provide acceptable reliability and linear relationship of k_*RFD–SF*_ and k_*RFR–SF*_ (main outcome measures). The major limitation of the previously conducted methodological studies is the small number of study participants. Only up to 15 participants were included in previous methodological studies investigating RFD-SF (Bellumori et al., 2011; Djordjevic and Uygur, 2017) and RFR-SF related measures (Mathern et al., 2019). Furthermore, k_*RFR–SF*_ has been investigated in only two studies even though it has been shown to be a sensitive measure for detection of neuromuscular impairments (Mathern et al., 2019), which was recently confirmed in knee osteoarthritis (Šarabon et al., 2020a) and multiple sclerosis patients (Uygur et al., 2020). There is no evidence yet about the associations between k_*RFD–SF*_ and k_*RFD–SF*_, which could provide valuable information on the fundamental understanding of force production and force relaxation in humans.

Based on the previous findings, the aims of our study were as follows. First, to find the smallest number of rapid isometric force pulses at different force amplitudes that is still valid and reliable for k_*RFD–SF*_ and k_*RFR–SF*_ calculation [intraclass correlation coefficient (ICC_2_,_1_) ≥ 0.95, coefficient of variation (CV) < 5%], compared to the standard protocol. We hypothesized that a twice smaller number of pulses would still provide a comparable and valid k_*RFD–SF*_ and k_*RFR–SF*_ calculation. Second, to introduce a new outcome measure from regression line – TP_*RFD*_ and TP_*RFR*_, respectively – and to assess its reliability. We hypothesized that the newly introduced outcome measures would yield valid results in the reduced protocol, while the opposite would be shown for y-intercept. Third, to investigate differences and associations between the coinciding k_*RFD–SF*_ and k_*RFR–SF*_. We hypothesized that associations would be moderate to large (negative), while the absolute values of the outcome measures would be significantly higher for k_*RFD–SF*_ than k_*RFR–SF*_.

## Materials and Methods

### Participants

A total of 336 (221 male and 115 female) participants (basketball, soccer, tennis players, long-distance runners, and students of Faculty of Sports) volunteered to participate in the study (Table 1). The inclusion criteria were at least 2 training sessions per week in the last year and a minimum of 3 years of training history. Participants with lower limb injuries, neurological disorders and low back pain in the past 6 months were excluded from the study. Leg side preference was determined by asking participants: “Which leg do you prefer when performing unilateral jumping movements?” The reliability and validity were first verified on a subset of participants (Table 1, 31 males and 9 females). In reliability group preferred and non-preferred legs were equally represented. All the participants (or their parents/guardians – in case participants were under the age of 18) were informed about the testing procedures and provided an informed consent prior to study participation. All the participants were asked to avoid intense physical activities at least 48 h prior to testing. Slovenian Medical Ethics Committee (approval no. 0120-99/2018/5) approved the experiment which was conducted according to the Declaration of Helsinki guidelines.

**TABLE 1:** Characteristics of the participants.

Group	Group	*N*	Age (years)	Body height (cm)	Body mass (kg)	BMI (kg/m^2^)	Left preferred (*n*)	Right preferred (*n*)	Training history (years)
Reliability	Male	31	16.7 ± 1.2	182.5 ± 9.2	75.2 ± 12.1	22.5 ± 2.6	31	9	7.8 ± 2.5
	Female	9	19.3 ± 8.5	178.6 ± 9.9	70.8 ± 11.5	22.1 ± 2.4	222	114	8.6 ± 7.0
Long-distance runners	Male	28	31.0 ± 9.4	182.2 ± 5.8	77.9 ± 6.6	23.5 ± 1.0	12	16	12.1 ± 8.9
	Female	15	34.6 ± 11.1	166.6 ± 8.1	60.4 ± 6.9	21.8 ± 2.1	8	7	7.5 ± 4.1
Basketball	Male	79	16.6 ± 1.1	188.1 ± 7.8	79.1 ± 10.6	22.3 ± 2.3	65	14	7.2 ± 2.3
	Female	40	16.9 ± 1.6	175.3 ± 5.8	70.8 ± 9.6	23.0 ± 2.8	37	3	6.8 ± 2.5
Students	Male	27	19.6 ± 0.4	182.9 ± 5.7	76.4 ± 8.5	22.8 ± 1.9	10	17	8.4 ± 3.7
	Female	25	19.7 ± 0.7	166.9 ± 6.0	59.9 ± 7.8	21.4 ± 2.1	11	14	8.6 ± 4.9
Tennis	Male	50	18.2 ± 14.9	177.1 ± 8.4	67.1 ± 11.0	21.3 ± 2.4	36	14	11.0 ± 15.2
	Female	35	16.3 ± 2.6	169.5 ± 5.6	61.8 ± 7.8	21.5 ± 2.1	24	11	7.9 ± 3.5
Soccer	Male	37	16.7 ± 1.0	179.7 ± 5.6	68.6 ± 8.4	21.2 ± 1.9	19	18	9.6 ± 2.1
	All	336	19.3 ± 8.5	178.6 ± 9.9	70.8 ± 11.5	22.1 ± 2.4	222	114	8.6 ± 7.0

### Study Design, Tasks, and Procedures

**FIGURE 1:**
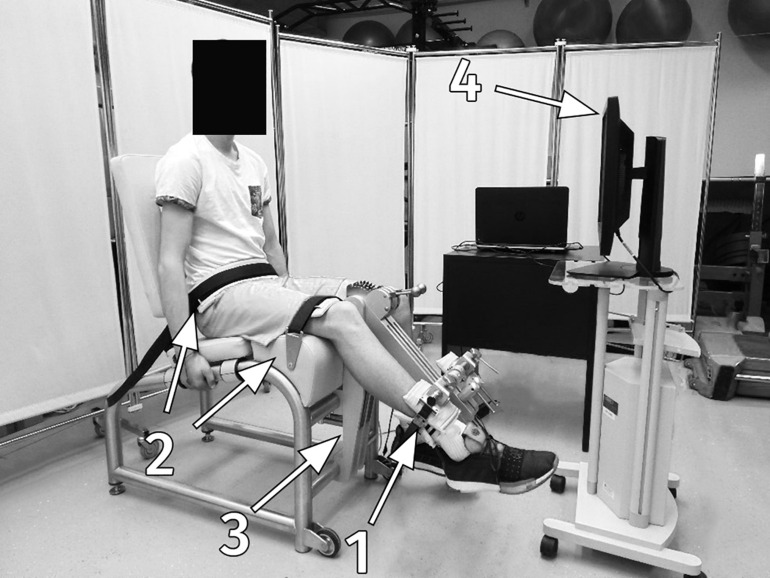
Measurement set-up; the subject in the isometric knee dynamometer: 1, a minimally padded shank support; 2, rigid straps for the knee and pelvis fixation; 3, a strain gage force sensor; 4, a monitor with visual feedback.

In the cross-sectional study, participants in the reliability group performed knee extension maximal isometric voluntary contraction and the standard RFD-SF/RFR-SF protocol with the randomly selected leg (preferred or non-preferred). Although we measured torque in our study, we will use the term force to maintain consistency with previous literature. During the testing protocol, participants were seated in the chair of an isometric knee dynamometer (S2P, Science to Practice, ltd., Ljubljana, Slovenia; Figure 1; Sarabon et al., 2013). Measurements were performed at 60° of the knee flexion (full knee extension represents 0°) and hips at 100°. The knee axis was aligned with the axis of the dynamometer’s lever arm. The minimally padded shank support was adjusted for each participant approximately 2 cm above the lateral malleolus. Good hip and knee fixation was ensured with rigid straps over the pelvis and knee. As part of accommodation, each participant performed two graded submaximal contractions at 50, 75, and 90% of the self-estimated maximal voluntary effort. After 3 min of rest, participants performed three maximal voluntary knee extensions with a 30-s rest in between. They were instructed to gradually increase their torque and sustain maximal force for 3–5 s which was used to determine knee extension peak force. The participants then performed an RFD-SF/RFR-SF familiarization protocol consisting of 15–20 submaximal explosive contractions and relaxations performed at different submaximal intensities or until they could perform voluntary force pulses as instructed. Participants were instructed to produce isometric knee extension as quickly as possible and to relax the muscles immediately afterward. After familiarization, each participant performed 25–30 explosive isometric contractions at four different submaximal levels (20, 40, 60, and 80% of previously determined maximal voluntary force), making for 100–120 contractions altogether. The target level of force was presented on a computer screen in front of the participant as a horizontal line on a graph. Visual feedback on the amount of force the participant had generated during the pulse was also provided on the screen, while participants were instructed to apply a level of force matching (about) the horizontal target force level during each pulse. Subjects were instructed to focus more on explosive performance rather than only trying to match force levels, as previously suggested (Gordon and Ghez, 1987). There was a 60-s rest between two consecutive submaximal levels. In the reliability group, each participant performed approximately 100–120 fast isometric contractions as previously suggested in several studies (Bellumori et al., 2011; Djordjevic and Uygur, 2017; Mathern et al., 2019). Explosive pulses were cued by experienced examinator with the verbal command at approximately 4–5 s intervals. Subsequently, all participants performed the reduced RFD-SF/RFR-SF protocol based on the results of the reliability group.

### Data Processing and Outcome Measures

The force transducer (Bending Beam Load Cell 1-Z6FC3, Darmstadt, Germany) signals from the knee dynamometer were sampled at 1000 Hz by a custom-made LabView 2015 routine (National Instruments Corp., Austin, TX, United States). The raw signal data were filtered with a low-pass filter (Butterworth 2nd order) with a 5-Hz cut-off frequency (Djordjevic and Uygur, 2017). Data were then analyzed in another adjusted custom-made LabView 2015 (National Instruments Corp., Austin, TX, United States) routine. The program routine automatically placed two cursors on the time derivate curve corresponding to peak RFD and peak RFR of each of the acquired force pulses. An additional cursor was placed on the force curve and depicted the peak force. All signals were manually inspected to verify correct cursor placement. The magnitudes and positions of peak force, RFD and RFR were recorded for each pulse (Mathern et al., 2019). The regression parameters were used as a dependent variable. They were obtained from the relationship between the peak torque and corresponding peak RFD and peak RFR. This relationship was used for calculation of the k_*RFD–SF*_ (/s) and k_*RFR–SF*_ (/s) of the regression line (main dependent outcome measures of interest). Other linear regression parameters, r^2^ (r^2^_*RFD*_, r^2^_*RFR*_), y-intercept [y-int_*RFD*_ (%MVC/s), y-int_*RFR*_ (%MVC/s)] and theoretical peak RFD/RFR [TP_*RFD*_ (%MVC/s), TP_*RFR*_ (%MVC/s)] were calculated for each participant. TP_*RFD*_ and TP_*RFR*_ represent the newly introduced outcome measures determined by linear interpolation for each participant’s regression line (*y* = *k* × *x* + *n*) where *x* = 100 (maximal theoretical peak RFR/RFR).

### Statistical Analysis

Statistical analysis was performed using R Statistical Software (version 4.0.3, R Core Team, R Foundation for Statistical Computing, Vienna, Austria). Descriptive statistics of the dependent variables are presented as means and standard deviations. Reliability and validity were first determined in a subset of participants (Table 2) to determine the smallest number of repetitions for k_*RFD–SF*_ and k_*RFR–SF*_ assessment, which would still provide excellent reliability (ICC_2_,_1_ ≥ 0.95, CV < 5%) in agreement with previously reported protocols (Bellumori et al., 2011; Djordjevic and Uygur, 2017). Although some of the previous studies considered CV < 15% to represent good validity (Staehli et al., 2010; Šarabon et al., 2020a), we used a more stringent criterium of CV < 5%. The ICC_2_,_1_ values were interpreted according to Koo and Li (2015). Typical error (TE) (Hopkins, 2000) and CV were also calculated, with CV expressed as TE relative to the mean of the protocols (within-subject standard deviation method). Comparative quantitative assessment of the protocol differences was performed by calculating Bland–Altman statistics (Bland and Altman, 1986) and drawing plots. Correlations between k_*RFD–SF*_ and k_*RFR–SF*_ were assessed by Pearson’s correlation coefficients and interpreted according to Hopkins et al. (2009) (0.00–0.19 trivial; 0.20–0.29 small; 0.30–0.49 moderate; 0.50–0.69 large; 0.70–0.89 very large; 0.90–0.99 nearly perfect; 1.00 perfect). Paired samples *t*-test was used for identifying differences between k_*RFD–SF*_ and k_*RFR–SF*_ and furthermore between TP_*RFD*_ and TP_*RFR*_. Because k_*RFR–SF*_ and TP_*RFR*_ are calculated from the descending part of the isometric pulse and have a negative sign, absolute numerical values were used for these outcome measures when performing the *t*-test. Cohen’s d effect size (*d*) was used to quantify the magnitude of the differences, using the following interpretation: negligible (<0.2), small (0.2–0.5), moderate (0.5–0.8) and large (>0.8) (Cohen, 1988). Significance level was set at *p* < 0.05 (two-tailed).

**TABLE 2:** Descriptive statistics, validity measures, and Bland–Altman statistics of rate of force development scaling factor (RFD-SF) and rate of force relaxation scaling factor (RFR-SF) outcome measures based on the standard and the reduced measurement protocol (*n* = 40).

Outcome measures	Mean ± SD (standard)	Mean ± SD (reduced)	ICC_2_,_1_ (95% CI)	CV%	TE
k_*RFD–SF*_ (/s)	8.98 ± 1.1	9.05 ± 1.1	0.97 (0.95, 0.98)	2.1	0.19
k_*RFR–SF*_ (/s)	−7.75 ± 1.5	−7.71 ± 1.6	0.96 (0.94, 0.98)	3.9	0.30
y-int_*RFD*_ (%MVC/s)	24.72 ± 29.4	24.01 ± 27.2	0.94 (0.90, 0.97)	*	6.85
y-int_*RFR*_ (%MVC/s)	−18.48 ± 46.3	−22.49 ± 48.2	0.94 (0.90, 0.96)	*	11.30
r^2^_*RFD–SF*_	0.97 ± 0.02	0.97 ± 0.02	0.84 (0.74, 0.90)	0.9	0.01
r^2^_*RFR–SF*_	0.89 ± 0.10	0.88 ± 0.12	0.93 (0.89, 0.96)	3.2	0.03
TP_*RFD*_ (%MVC/s)	922 ± 87.4	929 ± 89.2	0.98 (0.96, 0.99)	1.3	12.35
TP_*RFR*_ (%MVC/s)	−794 ± 125.5	−794 ± 133.1	0.98 (0.96, 0.99)	2.6	20.48

Standard, measurement calculation based on the standard protocol (100–120 pulses); reduced, measurement calculation based on the reduced protocol (36 pulses); ICC_2_,_1_ (95% CI), intraclass correlation coefficient (95% confidence interval); CV%, coefficient of variation; TE, typical error; k_*RFD–SF*_, slope of rate of force development scaling factor; k_*R*__*F*__*R–SF*_, slope of rate of force relaxation factor; y-int_*RFD*_, y-intercept of RFD-SF; y-int_*RF*__*R*_, y-intercept of RFR-SF; r^2^_*RFD–SF*_, linearity of regression line for RFD-SF; r^2^_*RFR–SF*_, linearity of regression line for RFR-SF; TP_*RFD*_, theoretical peak rate of fore development; TP_*RFR*_, theoretical peak rate of force relaxation. *CV not reported for two y-intercept outcome measure as it contains both positive and negative values.

Prior to participant recruitment, an *a priori* power calculation was performed. A sample size of 12 was required to achieve a power of 0.8 (α = 0.05, two-tailed) for the reliability analysis. The calculation was based on the sample size needed for the ICC_2,1_ to adequately detect a significant difference at a value of 0.95 (excellent reliability) (Zou, 2012). To test the third hypothesis at a power level of 0.80 (α = 0.05, two-tailed), a sample size of 149 and 199 was required for association analysis and paired samples *t*-test (using Cohen’s *d* = 0.2), respectively.

## Results

### Reliability of the Reduced Protocol

**FIGURE 2:**
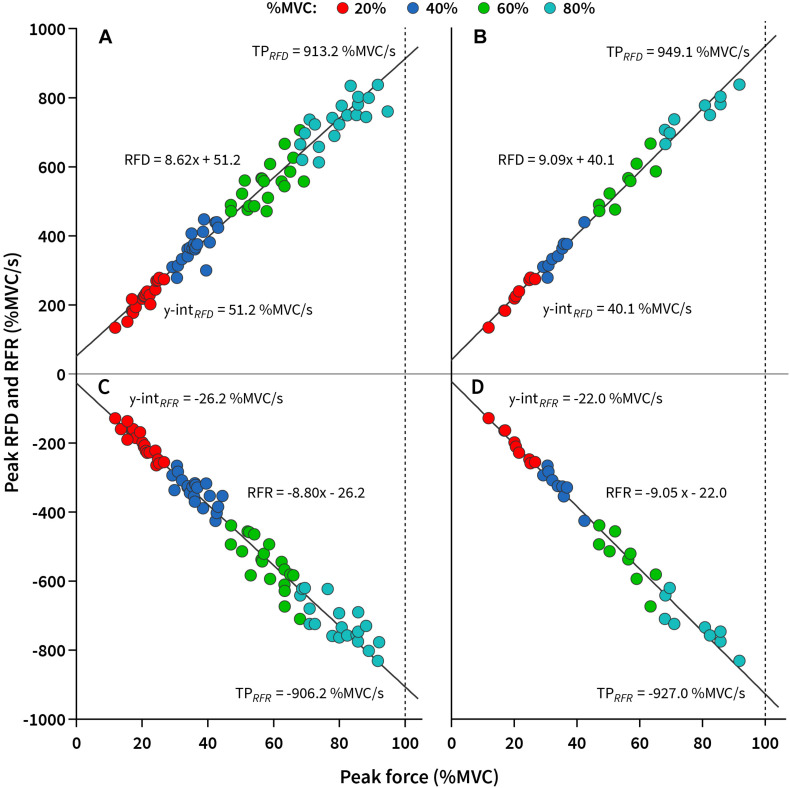
The slope of rate of force development/relaxation scaling factor (k_*RFD–SF*_/k_*RFR–SF*_) of a representative subject interpolated to a data scatter. **(A)** k_*RFD–SF*_ calculated based on the standard protocol (100–120 pulses). **(B)** k_*RFD–SF*_ calculated based on the reduced protocol (36 pulses). **(C)** k_*RFR–SF*_ calculated based on the standard protocol (100–120 pulses). **(D)** k_*RFR–SF*_ calculated based on the reduced protocol (36 pulses). y-int_*RFD*_, y-intercept of RFD-SF; y-int_*RF*__*R*_, y-intercept of RFR-SF; TP_*RFD*_, theoretical peak rate of fore development; TP_*RFR*_, theoretical peak rate of force relaxation.

The mean value during knee extension MVC for reliability group was 185.9 ± 54.8 Nm. Excellent relative (ICC_2_,_1_ ≥ 0.95) and absolute reliability (CV < 5%; based on our conservative criteria) were obtained for k_*RFD–SF*_ when seven pulses were used at each submaximal force amplitude (20, 40, 60, and 80% of MVC; 28 pulses in total) compared to the standard RFD-SF protocol (100–120 pulses in total). Meanwhile, the same reliability conditions were met for k_*RFR–SF*_ when nine pulses were used at each submaximal force amplitude (36 pulses in total). To meet the reliability criteria for calculating the regression line for the two main outcome measures (k_*RFD–SF*_, k_*RFR–SF*_) we used nine pulses for further calculation. Regression lines of a representative subject interpolated to a data scatter of the standard RFD-SF/RFR-SF protocol (100–120 pulses) and of the reduced protocol (36 pulses) are shown in Figure 2.

**FIGURE 3:**
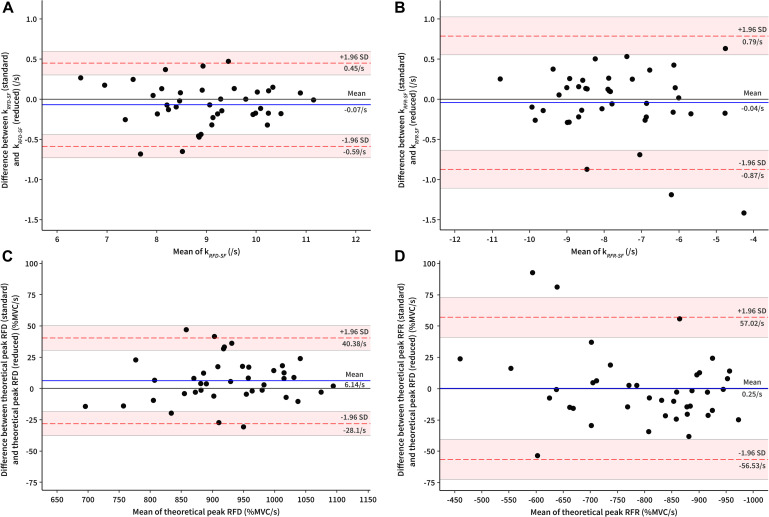
Bland–Altman plots depicting standard and reduced protocol mean values and differences for **(A)** k_*RFD–SF*_, **(B)** k_*RFR–SF*_, **(C)** theoretical peak rate of force development (TP_*RFD*_), and **(D)** theoretical peak rate of force relaxation (TP_*RFR*_). The solid line represents the mean bias and the dashed lines represent the limits of agreement for mean bias (shaded area represents 95% confidence intervals of the limits of agreement). k_*RFD–SF*_, slope of rate of force development scaling factor; k_*RFR–SF*_, slope of rate of force relaxation factor.

Validity and reliability statistics showed good agreement between the standard (100–120 pulses) and the reduced (36 pulses) protocols for all observed outcome measures. CV was not reported for both of the y-intercept outcome measures, as it contains positive and negative values (Shechtman, 2013; Table 2). Moreover, high linearity of regression line was calculated in both cases (Table 2). The Bland–Altman plots of k_*RFD–SF*_ and k_*RFR–SF*_ showed no systematic bias as most of the values were within the 95% limits of agreement (Figures 3A,B). Moreover, similar was shown for TP_*RFD*_ and TP_*RFR*_, which is demonstrated in Figures 3C,D.

### Differences and Associations Between RFD-SF and RFR-SF

The mean value during knee extension MVC for all participants was 198.6 ± 59.6 Nm. When all participants (*n* = 336) were evaluated using the reduced protocol our results showed significant large negative associations between k_*RFD–SF*_ (8.86 ± 1.0/s) and k_*RFR–SF*_ (−8.03 ± 1.3/s) (*r* = −0.502, *p* < 0.001). Similar associations were found between TP_*RFD*_ (920 ± 75.6 %MVC/s) and TP_*RFR*_ (−824 ± 106.0 %MVC/s) (−0.546, *p* < 0.001). Moreover, mean y-int_*RFD*_ was 33.83 ± 32.6 %MVC/s, while mean y-int_*RFR*_ was −21.57 ± 39.2 %MVC/s. Excellent regression line linearity was calculated for k_*RFD–SF*_ (r^2^_*RFD–SF*_ = 0.97 ± 0.03) and k_*RFR–SF*_ (r^2^_*RFR–SF*_ = 0.90 ± 0.09).

Paired samples *t*-test was performed for the main outcome measures of interest (k_*RTD–SF*_ and k_*RFR–SF*_) and for the newly introduced outcome measures. Our results showed significantly large differences in favor of k_*RFD–SF*_ compared to k_*RFR–SF*_ (*p* < 0.001, *d* = 0.72) and TP_*RFD*_ compared to TP_*RFR*_ (*p* < 0.001, *d* = 0.99).

## Discussion

The purpose of the present study was to verify the reduced protocol for calculating RFD-SF and RFR-SF related measures, to introduce new outcome measures (theoretical peak RFD/RFR, TP_*RFD*_, and TP_*RFR*_) and investigate differences and associations between k_*RFD–SF*_ and k_*RFR–SF*_. Our results revealed: (1) excellent reliability of the reduced RFD-SF/RFR-SF protocol for k_*R*__*F*__*D–SF*_ and k_*RFR–SF*_ employing a total of 36 isometric pulses (2) acceptable reliability of the newly introduced outcome measure (TP_*RFD*_ and TP_*RFR*_); and (3) large negative associations between k_*RFD–SF*_ and k_*RFR–SF*_ and significantly higher absolute values for k_*RFD–SF*_ than for k_*R*__*F*__*R–SF*_.

Our results showed excellent reliability (ICC_2_,_1_ ≥ 0.95) and acceptable CV (<5%) of the reduced protocol for both main outcome measures (k_*RFD–SF*_ and k_*RFR–SF*_) when nine pulses were used at each submaximal force (36 pulses total). Similar results were calculated for r^2^_*RFD*_, r^2^_*RFR*_, TP_*RFD*_, and TP_*RFR*_, while y-int_*RFD*_ and y-int_*RFR*_ yielded high TE compared to its mean values and standard deviations (Table 2). These results allow us to introduce the reduced protocol of RFD-SF and RFR-SF, where only 36 pulses are required for a reliable and valid calculation of the outcome measures. This is comparatively less than the previous standard protocol, which recommends performing 100–120 isometric pulses (Bellumori et al., 2011; Mathern et al., 2019). Moreover, in agreement with previous studies, we confirmed the negligibility of the y-intercept as an outcome measure (Casartelli et al., 2014; Bellumori et al., 2017; Djordjevic and Uygur, 2017). Performing 100–120 rapid isometric pulses require participants to be attentive and maintain concentration for extended periods. Furthermore, such number of rapid isometric submaximal contractions may induce fatigue in participants with lower capabilities as it was shown that low-frequency fatigue can occur in knee extensors after performing 60 submaximal concentric contractions (Baptista et al., 2009). For this reason, the reduced protocol is more time effective and user friendly from a fatigue standpoint. Nevertheless, it is necessary to not disregard the fact that some isometric force pulses are not performed properly (e.g., slow muscle contraction, improper relaxation…) and are later excluded from the analysis. Consequently, we suggest performing approximately 60 isometric pulses for a valid and reliable k_*RFD–SF*_ and k_*RFR–SF*_ calculation.

Because of previous evidence showing y-intercept unreliability, the purpose of our study was also to introduce a new outcome measure (TP_*RFD*_ and TP_*RFR*_). This measure has proven to be a more reliable outcome measure that can provide more valuable information. Our results showed that the calculation of TP_*RFD*_ and TP_*RFR*_ provides excellent ICC_2_,_1_, acceptable CV and TE. Based on this, we can confirm our second hypothesis. TP_*RFD*_ and TP_*RFR*_ can provide us with information about the participant‘s maximal theoretical RFD and RFR based on regression line of each participant. This outcome measure reflects the participant’s maximal theoretical capacity as opposed to the y-intercept, which reflects the theoretical RFD/RFR in cases where the peak force is equal to zero. This could be especially useful in people with different knee pain problems, where performing maximal contractions should be avoided because of pain. It has already been already shown that k_*RFD–SF*_ and k_*RFR–SF*_ can be assessed in knee osteoarthritis patients using only lower submaximal force values (Šarabon et al., 2020a). With the introduction of TP_*RFD*_ and TP_*RFR*_, additional outcome measures regarding neuromuscular quickness can be provided without subjecting participants to very high forces.

Our third aim was to compare differences and associations between k_*RFD–SF*_ and k_*RFR–SF*_ in a larger sample. To date, most of the studies have investigated k_*RFD–SF*_ of different muscle groups (Bellumori et al., 2013; Casartelli et al., 2014; Šarabon et al., 2020b; Smajla et al., 2020), while there are only two studies investigating k_*RFR–SF*_ on a smaller number of participants (Mathern et al., 2019; Šarabon et al., 2020a). None of these studies have investigated the associations between k_*RFD–SF*_ and k_*RFD–SF*_, or more specifically, associations between the ability for rapid force production and relaxation during different submaximal contractions. Our results showed that there is a large negative association between k_*RFD–SF*_ and k_*RFR–SF*_ (*r* = −0.502, *p* < 0.001) and significantly higher absolute values of k_*RFD–SF*_ compared to k_*RFR–SF*_ (*p* < 0.001, *d* = 0.72) in our young healthy participants. Similar results were seen when TP_*RFD*_ and TP_*RFR*_ were evaluated (*r* = −0.546, *p* < 0.001) (*p* < 0.001, *d* = 0.72). Based on this, we can confirm our third hypothesis. It is worth noting, that the associations between k_*RFD*__–SF_–k_*RF*__*R*__–SF_ and TP_*R*__*F*__*D*_–TP_*RFR*_ are negative, since k_*RFR–SF*_ and TP_*R*__*F*__*D*_ are calculated for the descending part of the isometric pulse and the values of k_*RFR–SF*_ and TP_*RFR*_ have a negative sign. Our results indicate that the ability for rapid force production and relaxation during isometric submaximal pulses is moderately associated, while force production during submaximal contractions is higher compared to force relaxation. Significantly higher k_*RFD–SF*_ and TP_*RFD*_ compared to k_*RFR–SF*_ and TP_*RFR*_ may be explained by different neuromuscular mechanisms. The k_*RFD–SF*_ mainly depends on neuromuscular activation mechanism (Folland et al., 2014) such as initial double discharges and high firing rates (Van Cutsem et al., 1998; Klass et al., 2008), whereas k_*RFR–SF*_ mostly depends on intrinsic properties of the muscle (Mathern et al., 2019). This is one of the reasons that both outcome measures should be assessed for a comprehensive neuromuscular assessment. Force relaxation is not investigated often, despite being functionally as important as quick force generation (Suzovic et al., 2008). It has been shown that muscle force relaxation is slower in young adults (Molenaar et al., 2013), people with Parkinson’s disease (Robichaud et al., 2005) and people with multiple sclerosis during stimulated tetanic force (Ng et al., 2004). However, methods utilizing maximal force production or various protocols of neuromuscular electrical stimulation are more invasive for such evaluation. On this basis, the reduced RFD-SF and RFR-SF protocol can be suggested as a practical and useful tool for neuromuscular testing in healthy individuals and participants with various impairments.

There are a few limitations of our study. Our sample size was not gender-balanced in the reliability group and the group where all participants were included. Possible gender influence on k_*RFD–SF*_ and k_*RFR–SF*_ merits further investigation by employing a gender balanced sample. Moreover, the long-distance runners’ group was significantly older compared to other groups, However, we included them in the analysis as previous studies have shown excellent reliability of RFD-SF and RFR-SF measurements in similar age groups (Mathern et al., 2019). Moreover, we evaluated only the participant’s preferred leg (when all participants were evaluated) although differences in neuromuscular quickness may exist between preferred and non-preferred leg. This also warrants investigation in future studies. Furthermore, despite the large sample size in our study, our conclusions can only apply to young, physically active and healthy individuals.

Brief submaximal actions are important for a wide range of functional movements (e.g., postural corrections and reaching) or in consecutive actions (contraction and relaxation) of different muscle groups (e.g., walking and running). Our results suggest that the reduced RFD-SF/RFR-SF protocol is reliable for quantifying neuromuscular abilities to quickly produce and relax muscle force during submaximal contractions. The advantage of the reduced protocol is the shorter assessment time and the reduction of the possible influence of fatigue. The introduction of TP_*RFD*_ and TP_*RFR*_ provides additional valuable information about participants maximal theoretical RFD/RFR, while this is not the case for the y-intercept. This can be useful for the assessment of maximal capacity in people with various impairments or pain problems where performing maximal contractions is not possible. Based on our results, we conclude that analyzing 36 isometric force pulses is sufficient for a valid and reliable RFD-SF and RFR-SF related measures calculation. However, we recommend performing approximately 60 pulses to ensure that there is a sufficient number of pulses available for the calculation.

## Data Availability Statement

The raw data supporting the conclusions of this article will be made available by the authors, without undue reservation.

## Ethics Statement

The studies involving human participants were reviewed and approved by the Slovenian Medical Ethics Committee (approval no. 0120-99/2018/5). Written informed consent to participate in this study was provided by the participants’ legal guardian/next of kin. Written informed consent was obtained from the minor(s)’ legal guardian/next of kin for the publication of any potentially identifiable images or data included in this article.

## Author Contributions

NŠ overviewed the measurement procedures and administration and conceptualized the idea. DS and JŽ collected the data. JŽ analyzed the collected data. DS wrote the manuscript. NŠ and JŽ finalized the manuscript. All authors contributed to the article and approved the submitted version.

## Conflict of Interest

NŠ was employed by the company S2P, Science to Practice, Ltd., Laboratory for Motor Control and Motor Behavior (Ljubljana, Slovenia). The remaining authors declare that the research was conducted in the absence of any commercial or financial relationships that could be construed as a potential conflict of interest.
